# Artesunate alleviates kidney fibrosis by restoring klotho protein and suppressing Wnt/β-catenin signalling pathway

**DOI:** 10.3389/fcell.2025.1688753

**Published:** 2025-11-13

**Authors:** Goran H. Mohammad, Julius Kieswich, Abhishek Kumar, Kieran McCafferty, Ramyangshu Chakraborty, Christoph Thiemermann, Magdi M. Yaqoob

**Affiliations:** 1 Centre for Translational Medicine and Therapeutics, William Harvey Research Institute, Barts and the London School of Medicine and Dentistry, Queen Mary University of London, London, United Kingdom; 2 Chemistry Department, College of Science, University of Sulaimani, Sulaymaniyah, Iraq; 3 Diabetic Kidney Disease Centre, Renal Unit, Barts Health National Health Service Trust, The Royal London Hospital, London, United Kingdom

**Keywords:** artesunate, kidney fibrosis, TGF-β, klotho, ferroptosis

## Abstract

Chronic kidney disease (CKD) is a global health concern that often progresses to renal failure and premature death. Regardless of etiology of CKD, kidney fibrosis is the main determinant of progressive CKD. Renal fibrosis is characterized by excessive collagen and extracellular-matrix (ECM) deposition, which impairs renal function with an irreversible loss of nephrons. Currently, there are no effective antifibrotic therapies to halt the progression of CKD to the end-stage-kidney-failure (ESKF). Artesunate has recently shown antifibrotic effects in various animal models, but its efficacy in renal fibrosis remains unexplored. In this study, the efficacy of artesunate was evaluated in a unilateral-ureteral-obstruction (UUO) mouse model and in primary human kidney fibroblasts (HKF). Mechanistic investigation including immunoblot analysis, immunohistochemistry, gene expression assay, enzyme-linked-immunosorbent assay (ELISA) and other tools were used to study the underlying molecular mechanisms of antifibrotic effects of artesunate. Results of this study showed that artesunate ameliorated multiple profibrotic pathways including transforming-growth-factor-beta (TGF-β) expression in UUO model and reduced profibrotic markers including alpha-smooth-muscle-actin (α-SMA), fibronectin, collagen I, and vimentin in both *in-vivo* and *in-vitro* models. Mechanistic studies indicated that artesunate treatment abrogated the TGF-β/SMAD pathway, restored klotho-protein expression and attenuated both PI3K/Akt and Wnt/β-catenin pathways. Additionally, artesunate inhibited cell-proliferation in UUO and induced ferroptosis in HKF cell culture. In conclusion, our study demonstrates that artesunate treatment abrogated fibroblast activation, attenuated canonical and non-canonical TGF-β pathways, inhibited cell proliferation in the UUO and selectively induced ferroptosis in HKF cell culture. These findings suggest that artesunate may have the potential to delay the progression of CKD and mitigate the development of kidney fibrosis, providing a promising direction for future therapeutic investigation.

## 1 Introduction

CKD is a global health problem with high co-morbidities and mortality. The disease has a serious risk to human health and often progress to renal failure and premature death (Sun et al., 2023; Kovesdy, 2022). Regardless of CKD etiology, kidney fibrosis represents the final pathway of renal demise and is characterised by excessive collagen deposition and ECM accumulation in the tubulointerstitium, causing renal impairment and irreversible nephrons loss (Hsieh et al., 2021; Andrikopoulos et al., 2019). The pathogenesis of kidney fibrosis is complex and not fully understood. However, renal injury, inflammation, fibroblast activation and proliferation, growth factors and dysregulation of fibrogenic signalling are thought to be involved (Antar et al., 2023; Reiss et al., 2024).

TGF-β and SMAD family signalling are master regulators of renal fibrosis through canonical and non-canonical TGF-β/SMAD pathways (Ahuja and Zaheer, 2024; Lee et al., 2024). In the canonical pathway, TGF-β binds to the TGF-β receptors and triggers SMAD2/3 phosphorylation, then SMAD4 (co-SMAD) directly interacts with phospho-SMAD2/3 and forms a SMAD2/3/4 complex and then translocate to the nucleus to induce and regulate profibrotic genes transcription (Ahuja and Zaheer, 2024; Park and Yoo, 2022).

The PI3K/Akt/mTOR pathway is a non-canonical TGF-β signalling pathway that contributes to the induction of epithelial to mesenchymal transition (EMT) and renal fibrosis (Derynck et al., 2014; Di Gregorio et al., 2020). As previously reported, TGF-β activates the PI3K/Akt pathway through direct interaction between the p85-subunit of PI3K and TGF-β receptor II. Upon activation, PI3K phosphorylates its downstream effector, Akt. The PI3K/Akt pathway then activates another downstream effector of the mTOR protein, which is a key regulator of protein synthesis via phosphorylation of S6-ribosomal protein (S6) and eukaryotic initiation factor 4E-binding-protein-1 (4E-BP1). Phosphorylation of S6 and 4E-BP1 by mTOR enhances translational capacity, induces cell proliferation, protein synthesis, EMT induction and renal fibrosis (Andrikopoulos et al., 2019; Liu, 2011; Wang et al., 2024).

The Wnt/β-catenin pathway is another downstream of TGF-β/SMAD and plays an important role in renal fibrosis and is significantly upregulated during kidney fibrosis (Wang et al., 2024; Song et al., 2024). During renal fibrosis, the upregulation of Wnt expression activates the Wnt/β-catenin pathway through the binding of Wnt ligands to Frizzled receptors and LRP5/6 co-receptors. This interaction inhibits the β-catenin destruction complex (composed of APC, Axin, GSK-3β, and CK1), leading to the stabilisation and accumulation of β-catenin in the cytoplasm which then facilitates the translocation into the nucleus to interact with TCF/LEF transcription factors that regulate the expression of Wnt-target genes (Wang et al., 2024; Song et al., 2024; Tan et al., 2014). Previous studies have reported the crosstalk between TGF-β/SMAD and Wnt/β-catenin pathways. TGF-β-induced fibrosis can be exacerbated by activation of Wnt/β-catenin pathway, and contributes to the fibroblast activation, ECM and collagen deposition which further aggravates renal fibrosis (Vallée et al., 2017; Gumede et al., 2024). Additionally, klotho is an anti-aging protein, functioning as an antagonist to the Wnt/β-catenin pathway and often downregulated in CKD. Previous studies have reported the protective role of klotho against kidney injury and fibrosis, revealing that the loss of klotho promotes the Wnt/β-catenin pathway, exacerbates kidney injury and accelerates the progression of CKD to ESKF and kidney fibrosis (Zhou et al., 2013; Yuan et al., 2022; Zhou et al., 2022). Indeed, inflammation is another key contributor to the development and progression of renal fibrosis through a complex interplay of cellular and molecular mechanisms. Pro-inflammatory cytokines and growth factors stimulate the fibroblast proliferation and differentiation into myofibroblasts, leading to the excessive ECM production, collagen deposition and tissue scarring (Kuang et al., 2018; Lv et al., 2018; Meng, 2019).

Currently, there are no effective antifibrotic treatments to halt the progression of CKD to the ESKF. Several therapeutic strategies designed to inhibit TGF-β expression, alleviate TGF-β signalling and abrogate the fibroblast activation; however, none of these strategies were translated clinically as demonstrated by unsuccessful clinical trials (Reiss et al., 2024; Park and Yoo, 2022; Zhao et al., 2022). Another potential approach to prevent tissue fibrosis is the inhibition of fibroblast proliferation and transdifferentiation into myofibroblast. Artemisinin is an active ingredient of *Artemisia annua-L*-plant (Chinese herbal medicinal plant), artesunate is one of the Artemisinin derivatives and a first line anti-malaria drug. Artesunate has shown anti-fibrotic effect in multiple animal models, including ocular (Liu et al., 2023a), liver (Zhang et al., 2020a) and epidural fibrosis (Wan et al., 2019) by inhibiting fibrotic signalling and inducing ferroptosis. Despite these promising findings, the therapeutic efficacy of artesunate in CKD and renal fibrosis remains unexplored. This study investigates the renoprotective potential of artesunate and elucidates the molecular mechanisms and signalling pathways, particularly those associated with fibrogenesis, oxidative stress, and ferroptosis, that may mediate its antifibrotic effects in the kidney.

Ferroptosis is a form of regulated cell death characterized by the accumulation of lipid peroxides and iron-dependent oxidative damage. It is driven by the failure of the glutathione dependent antioxidant defence system, particularly the depletion of glutathione and the inactivation of nuclear factor erythroid-2 related factor-2 (Nrf2), glutathione peroxidase-4 (GPX4) and ferroptosis suppressor protein-1 (FSP1). This leads to the unchecked accumulation of reactive oxygen species (ROS) and lipid peroxides, ultimately causing cell death (Feng et al., 2023; Zhang et al., 2022).

The aim of the present study is to explore the potential therapeutic effect of artesunate on UUO induced renal fibrosis and its potential to prevent the progression of CKD to kidney fibrosis. In addition, we investigate key fibrogenic signalling pathways, including TGF-β/SMAD, PI3K/Akt/mTOR and WNT/β-catenin, to elucidate the underlying molecular mechanisms.

## 2 Materials and methods

### 2.1 Animals experiments

All animal experiments were conducted in accordance with the United Kingdom Home Office Animals 1986 Scientific Procedures with approval granted by our local ethical committee (Project License number: P73DE7999). Male C57BL/6J mice weighing between 20 and 25 g at 6–8 weeks of age were purchased from Charles River United Kingdom Ltd. (Margate, Kent, United Kingdom) and were used in this study. The mice underwent UUO surgery according to the previous published methods (Chevalier et al., 2009; Martínez-Klimova et al., 2019). Briefly, mice were anesthetised with 2% isoflurane and the abdominal cavity was exposed using midline laparotomy. Subsequently, the left ureter was isolated and tied off 0.5 cm from the pelvis using a sterile 5-0 silk-braided suture, whilst the right ureter was left unclamped. All incisions were closed using a 5-0 Proline suture. The sham operation mice underwent the same surgery but without the ligation of the ureter. Following UUO surgery for a period of 10 days in total, artesunate (100 mg/kg) or vehicle (5% NaHCO3) was administered daily by intraperitoneal injection (100 μL/animal) (Liu et al., 2023b). Artesunate (Thermo Scientific, Fisher Scientific, Loughborough, Leicestershire, United Kingdom) solution originally was prepared for *in vivo* injection by dissolving in 5%NaHCO3 (VWR Chemicals BDH, Lutterworth, Leicestershire United Kingdom) at a concentration of 100 mg/mL. After surgery, the mice divided into four groups. (1) Sham-treated with vehicle (n = 6 mice), (2) Sham-treated with artesunate (n = 6 mice), (3) UUO-treated with vehicle (n = 8 mice), (4) UUO-treated with artesunate (n = 8 mice).

At the end of the experiment, the mice were sacrificed, and both kidneys and blood were collected for further analysis. Both kidneys from each animal were cut in half longitudinally. One-half of each kidney was snap-frozen in liquid nitrogen and subsequently stored at −80 °C for Western blotting analysis. The other half was fixed with 10% Formalin (Merck Life Science United Kingdom Ltd, Gillingham, Dorset, United Kingdom) for 16 h at 4 °C then transferred into a 70%v/v ethanol solution for a further 24 h and was then subsequently embedded in paraffin for immunohistochemistry. Blood was immediately centrifuged at 10,000 rpm at 4 °C for 15 min and the serum was separated and collected in fresh Eppendorf tubes and stored at −80 °C for later analysis.

### 2.2 Immunohistochemistry

Formalin fixed kidney tissues were embedded in paraffin and cut into 4 µm sections. Immunohistochemistry was performed as previously described (Mohammad et al., 2016; Dao et al., 2020). Briefly, kidney sections were pre-heated in an oven for 1 h at 60 °C, and then deparaffinised in xylene and hydrated through a series of graded ethanol concentrations (70%–95%). The sections were subjected to heat-mediated antigen retrieval with citrate buffer (pH 4.0) in an autoclave. Endogenous peroxidase activity was inhibited by 3% hydrogen peroxide for 20 min, followed by incubation with 3% normal horse blocking serum for 20 min. The sections were then incubated with primary antibody overnight at 4 °C for α-SMA, F4/80 and Ki-67 (Antibodies detail listed in Supplementary Table S1). After three washes with PBS containing 0.5% Tween 20, the sections were incubated with horseradish peroxidase (HRP)–conjugated secondary antibody for 30 min. Staining was visualized using the 3, 3′-diaminobenzidine (DAB) detection system kit. The sections were placed in haematoxylin for 2 min, then gently washed and mounted.

Staining with hematoxylin and eosin or sirius red were performed as previously described (35). Images of stained sections were captured using a NanoZoomer S210 Slide Scanner microscope. Ten images of kidney cortex were captured at 20x magnification per mouse and staining was quantified as percentage of total area using ImageJ 1.5t (National Institutes of Health). For Ki67 staining, positive nuclei per field of view were counted instead.

### 2.3 Western blot

Immunoblot analysis of kidney samples and human primary kidney cell lines were evaluated by Western blotting as previously described (Mohammad et al., 2021). Briefly, following protein extraction, protein concentration was measured by the Bicinchoninic Acid (BCA) assay kit (Thermo Scientific, Fisher Scientific, Loughborough, Leicestershire, United Kingdom) and 30 μg protein was run on a precast gel (NuPAGE Novex 4%–12% Bis-Tris 1.0 mm, 12 well gel, Invitrogen, Fisher Scientific) and transferred onto a 0.45 μm pore size Polyvinylidene fluoride (PVDF) membrane (Amersham, VWR). The membrane was blocked with 5% skim milk solution (OXOID, Merck Life Science Ltd., United Kingdom) and incubated with primary antibodies overnight at 4 °C (Antibodies detail listed in Supplementary Table S1). The membrane was then incubated with appropriate HRP-conjugated secondary antibodies either anti-mouse or anti-rabbit. The antigen antibody reaction was detected by enhanced chemiluminescence substrate (Amersham, VWR). GAPDH or β-actin antibody was used as a protein loading control. ImageJ software was used to determine relative band intensity in order to quantity protein expression.

### 2.4 Cell cultures

Primary human kidney fibroblasts (HKF) were purchased from DV Biologics and the human renal proximal tubule cells (HK2) were obtained from ATCC. HKF and HK2 cells were grown in DMEM and DMEM-F12 respectively, supplemented with 10% FBS, 1% penicillin/streptomycin (Merck) and the cells were maintained in humidified atmosphere of 20% O2, 5% CO2 at 37 °C as described previously (Andrikopoulos et al., 2019; Mohammad et al., 2016). To study the effect of artesunate on HKF and HK2 cell proliferation, the cells were depleted with serum free medium for 24 h, then were stimulated with 10 ng/mL TGF-β1 (Rockland, from Cambridge Bioscience, Cambridge United Kingdom), before treatment with different concertation of artesunate ranged from 0 to 120 µM. After 48 h treatment, the cells washed twice with ice cold PBS and lysed with RIPA buffer (EMD Millipore, Merck) containing protease (Merck) and phosphatase inhibitor cocktails (Roche, Merck). Protein quantity was measured using BCA method and stored at −80 °C for further analysis.

### 2.5 Cell viability assay

The cytotoxicity of artesunate treatment on HKF and HK2 cells were assessed by MTS assay (Promega United Kingdom Ltd, Southampton, Hampshire United Kingdom). The cells were seeded at a density of 5 × 103 cells per well, in 96 well plates in complete media and placed overnight at 37 °C in a humidified incubator. The following day, the media was replaced with fresh serum free media containing different concentrations of artesunate ranged from 0 to 300 µM. After 48 h incubation, cell viability was measured using MTS assay according to the manufactory instruction. The drugs’ half inhibitory concentration (IC_50_) was calculated by using GraphPad Prism Software (v10) from the dose-response curves of percentage of cell growth vs. drug concentration. Each artesunate treatment concentration was assayed in quadruplicates and the experiment was repeated at least three times.

### 2.6 ELISA

Pro-inflammatory cytokines in serum samples were evaluated using enzyme linked immunosorbent assay kit (ELISA). The concentration of IL-1β, IL-6 and TNF-α were measured in mice serum, according to the manufacturer’s instruction (IL-1β, Abcam), (IL-6, R&D systems), (TNF-α, Fisher Scientific). The optical density of each cytokine was measured with a microplate reader at 450 nm, then the concentration was calculated against a standard curve using GraphPad Prism software (v10).

### 2.7 Gene expression assay

Total RNA was isolated from kidney tissues, and HKF using the RNA extraction kit (Qiagen, Manchester United Kingdom) according to the manufacturer’s instruction. One microgram of extracted RNA was reverse transcribed to cDNA using a reverse transcription kit (Qiagen). Real-time PCRs were performed using SYBR Green master mix (BIO-RAD) using 10 ng of cDNA. The primers used for the target genes are listed in Supplementary Table S2. The relative gene expression was determined by normalising the expression of each gene to that of the β-Actin or GAPDH gene using the 2^−ΔΔCT^ method.

### 2.8 Statistics

Data are expressed as mean ± SEM. An unpaired Student’s t-test was used for comparison between two groups. For multiple comparisons, one- or two-way ANOVA with Tukey’s *post hoc* test was performed using GraphPad Prism v10. Statistical significance was set at p < 0.05.

## 3 Results

### 3.1 Artesunate reduced pro-fibrotic markers expression, ECM and collagen deposition in both *in-vivo* and *in-vitro* renal fibrosis models

**FIGURE 1:**
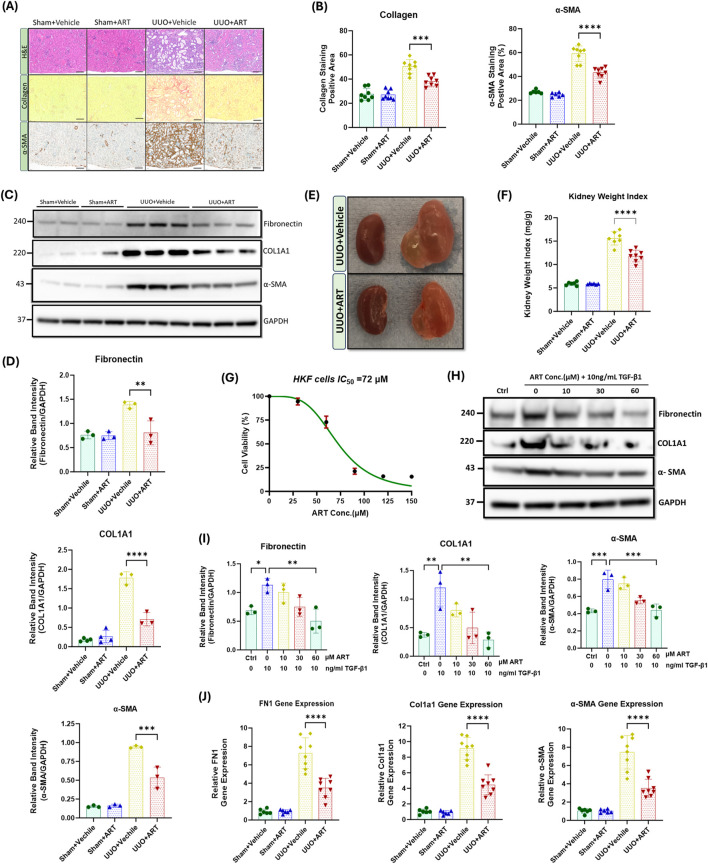
Artesunate treatment ameliorated expression of pro-fibrotic markers and reduced the deposition of ECM and collagen in both *in-vivo* and *in-vitro* renal fibrosis models. **(A)** Staining with Haematoxylin and Eosin (H&E), Sirius Red and immunostaining of kidney sections for α-SMA from obstructed (UUO; 10 days after surgery) or contralateral sham-operated kidneys (control) at 20x magnification (scale bar 50 µm). Animals received artesunate (100 mg/kg/day for 10 days) or vehicle as indicated. **(B)** Quantification of collagen deposition and positive α-SMA-stained area as percentage of total area (n = 8, one-way ANOVA, p ≤ 0.0001). **(C)** Western blot of whole-kidney lysates for fibronectin, collagen and α-SMA expression. PVDF membranes were subsequently stripped of antibodies and reprobed for GAPDH, which served as a protein loading control. **(D)** Relative band intensities of fibronectin, collagen and α-SMA expression were quantified and normalised against GAPDH using ImageJ software (n = 3, one-way ANOVA, p ≤ 0.0044). **(E)** Representative images of the obstructed (left) and contralateral kidney (right) in artesunate and vehicle treated group after 10 days post-UUO surgery. **(F)** Effect of artesunate and vehicle treatment on UUO kidney mass was represented by the kidney weight/body weight ratio (kidney weight ratio) (n = 8, one-way ANOVA, p < 0.0001). Primary human kidney fibroblasts (HKF) were grown in a complete DMEM medium and maintained in a humidified atmosphere incubator. To study the effect of artesunate on HKF cell-proliferation, the cells starved with serum free medium for 24 h and then stimulated with 10 ng/mL TGF-β1, before treatment with different concertation of artesunate ranged from 0 to 150 mM for 48 h. **(G)** The cytotoxicity of artesunate treatment on primary human kidney fibroblast cells was measured by MTS assay and the IC_50_ was calculated. **(H,I)** The expression level of fibronectin, collagen and α-SMA protein in response to different artesunate concentrations on HKF cells were assessed using Western blotting analysis and the blots were quantified by using ImageJ software and normalised to the GAPDH (n = 3, one-3way ANOVA, p ≤ 0.0347). **(J)** Representative of relative fibronectin, collagen and α-SMA gene expression from kidney of animals subjected to UUO, treated with artesunate and vehicle as indicated verses the control group (n = 8, one-way ANOVA, p < 0.0001). For all graphs, error bars represent the mean ± SEM of data from 3–8 animals per group. Statistical significance is indicated as ^ns^p≥0.05, *p < 0.05, **p < 0.01, ***p < 0.001, ****p < 0.0001.

To evaluate the potential therapeutic effect of artesunate, mice were treated with artesunate (100 mg/kg/day) for 10 days following UUO surgery. UUO-kidneys exhibited severe dilation, tubulointerstitial expansion and morphological changes. However, kidneys from artesunate-treated mice displayed significantly less dilation and fewer morphological abnormalities (Figures 1A,E). Additionally, kidney weight index was significantly higher in the UUO groups compared to the sham-groups. However, artesunate treatment significantly reduced the kidney weight index compared with that in the UUO vehicle-treated group (Figures 1E,F).

Upregulation of profibrotic markers such as collagen, α-SMA and fibronectin are a hallmark of kidney fibrosis. UUO-kidney sections staining showed that the artesunate-treated mice exhibited significantly less collagen deposition in the kidney interstitium and positive α-SMA staining was significantly reduced compared to the UUO-kidneys in vehicle-treated mice (Figures 1A,B). Western blotting analysis confirmed the effect of artesunate upon profibrotic markers expression which showed reductions of collagen deposition, α-SMA and fibronectin expression in UUO-kidneys by 60%, 43% and 42% respectively, compared to vehicle-treated UUO-kidneys (Figures 1C,D). Our *in-vitro* results further confirmed the therapeutic effect of artesunate with significantly reduced profibrotic markers present in renal fibroblast cell culture. HKF cells were stimulated with TGF-β in the presence or absence of artesunate. First, a dose response curve of artesunate was performed by MTS assay and IC_50_ was calculated as 72 μM (Figure 1G). Lower doses than the IC_50_ have been chosen for this study. Western blotting analysis revealed that artesunate reduced profibrotic markers expression in a dose-dependent manner in renal fibroblasts. At a concentration of 60 μM, artesunate significantly downregulated fibronectin, collagen, α-SMA, connective tissue growth factor (CTGF) and vimentin expression by 55%, 76%, 45%, 44% and 47% respectively and inhibited renal fibroblast proliferation (Figures 1H,I; Supplementary Figure S1).

Gene expression assay further confirmed the ability of artesunate to prevent the progression of kidney fibrosis. Artesunate treatment significantly reduced fibronectin, collagen, α-SMA, CTGF and vimentin gene expression in UUO-kidneys by 52%, 51%, 53%, 51% and 52% respectively (Figure 1J; Supplementary Figure S2).

Overall, these results demonstrate the ability of artesunate to supress the expression of pro-fibrotic markers and reduce ECM and collagen deposition in both *in-vivo* and *in-vitro* renal fibrosis models and to inhibit renal fibroblasts expansion and transdifferentiation into myofibroblasts.

### 3.2 Artesunate suppressed TGF-β expression and alleviated the activation of the TGF-β/SMAD pathway in UUO-kidneys and renal fibroblasts

TGF-β plays a central role in the development and progression of kidney fibrosis through canonical and non-canonical pathways. Consequently, we investigated the effect of artesunate treatment on TGF-β expression and the TGF-β/SMAD pathway.

**FIGURE 2:**
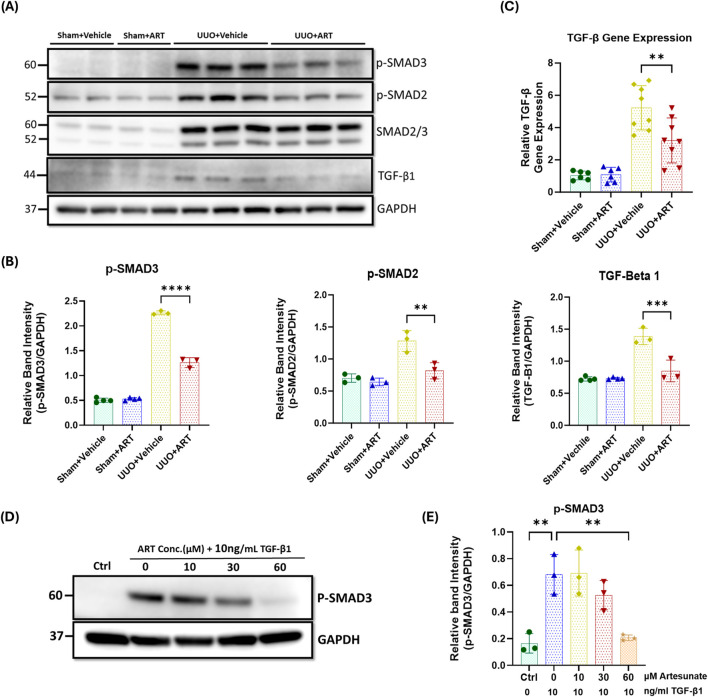
Artesunate administration reduced TGF-β expression and attenuated TGF-β/SMAD pathway in UUO kidneys and renal fibroblast cell culture. **(A,B)** Western blotting was performed to evaluate the expression level of TGF-β, p-SMAD3, p-SMAD2 and SMAD2/3 proteins in UUO kidneys and control group and ImageJ software was used to quantify the blots and normalised to GAPDH (n = 3, one-way ANOVA, p ≤ 0.0043). **(C)** mRNA level of TGF-β in UUO kidneys and control group were quantified by using quantitative polymerase chain reaction (qPCR) (n = 8, one-way ANOVA, p = 0.0053). **(D,E)** HKF cells were starved with serum free medium for 24 h and then stimulated with 10 ng/mL TGF-β1, before treatment with different concertation of artesunate ranged from 0 to 60 mM for 48 h. The cells were then lysed and the expression level of p-SMAD3 protein was determined by Western blotting, the blots were quantified by using ImageJ software and normalised to the GAPDH (n = 3, one-way ANOVA, p ≤ 0.0043). The data are presented as the mean ± SEM from 3–8 animals per group. Statistical significance is indicated as ^ns^p ≥ 0.05, *p < 0.05, **p < 0.01, ***p < 0.001, ****p < 0.0001.

Western blotting analysis revealed a dramatic upregulation of TGF-β expression and increased phosphorylation of both SMAD2 and SMAD3 in obstructed kidneys. However, artesunate administration effectively suppressed TGF-β expression and significantly reduced the SMAD2 and SMAD3 phosphorylation by 39%, 32% and 44%, respectively (Figures 2A,B). Gene expression assay confirmed the capability of artesunate to reduce TGF-β mRNA expression in UUO-kidneys. As shown in the Figure 2C, artesunate administration remarkably reduced TGF-β mRNA-expression by 39% in UUO-kidneys compared to the vehicle-treated group.

SMAD3 is a key downstream mediator of the TGF-β signalling, and a critical driver of the fibrotic process. It facilitates fibroblast differentiation into myofibroblasts and directly promotes the expression of fibrogenic genes (Yang et al., 2021; Zhao et al., 2016; Chen et al., 2014; Sun et al., 2013). Western blotting analysis showed a dramatic upregulation of phospho-SMAD3 in stimulated fibroblast cell culture, whilst artesunate treatment gradually reduced phospho-SMAD3 expression in a dose-dependent manner, restoring it to control levels at a concentration of 60 µM (Figures 2D,E).

Collectively, these results demonstrate the ability of artesunate to supress TGF-β expression and alleviate the activation of the TGF-β/SMAD signalling and repress the transcription of pro-fibrotic genes in UUO-kidneys and renal fibroblast cell culture.

### 3.3 Artesunate attenuated the PI3K/Akt pathway and reduced cell proliferation in UUO-kidneys and renal fibroblasts

**FIGURE 3:**
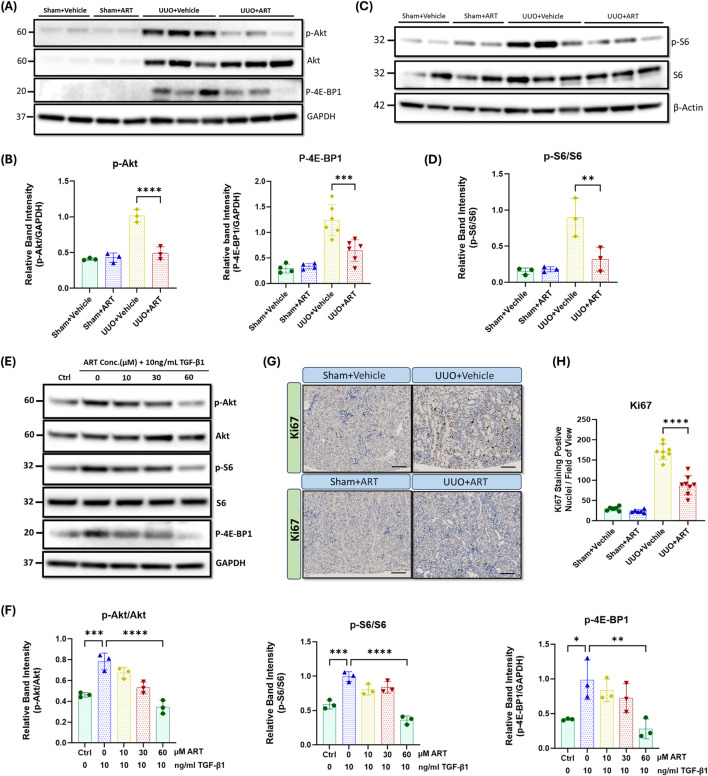
Artesunate administration ameliorated PI3K/Akt pathway and reduced cell-proliferation in UUO kidneys and renal fibroblast cell culture. **(A,B)** p-AKT, AKT and p-4E-BP1 proteins expression were evaluated in UUO kidneys and control group by using Western blotting analysis and ImageJ software was used to quantify the blots and normalised to GAPDH protein expression (n ≥ 3, one-way ANOVA, p ≤ 0.001). **(C,D)** Western blotting was performed to assess the expression level of p-S6 and S6 protein in UUO kidneys and control group and ImageJ software was used to quantify the blots and normalised to β-Actin (n = 3, one-way ANOVA, p = 0.0095). HKF cells were starved with serum free medium for 24 h and then stimulated with 10 ng/mL TGF-β1, before treatment with different concertation of artesunate ranged from 0 to 60 mM for 48 h. The cells were then lysed and the expression level of p-Akt, Akt, p-S6, S6 and p-4E-BP1 **(E, F)** proteins were determined by Western blot. ImageJ software was used to quantify the blots and p-Akt and p-S6 normalised to the total Akt and S6 protein respectively and p-4E-BP1 normalised to GAPDH (n = 3, one-way ANOVA, p ≤ 0.0062). **(G,H)** Representative UUO kidney and control kidney sections were immunostained with anti-Ki67 antibody and the number of Ki67 positive nuclei per field of view in UUO kidney and control kidney sections were quantified (20x magnification, scale bar 50 µm) (n = 8, one-way ANOVA, p < 0.0001). The data are presented as the mean ± SEM from 3–8 animals per group. Statistical significance is indicated as ^ns^p ≥ 0.05, *p < 0.05, **p < 0.01, ***p < 0.001, ****p < 0.0001.

To understand the molecular mechanisms underlying the inhibitory effect of artesunate in obstructive kidney and renal fibroblasts, we next investigated the PI3K/Akt/mTOR signalling pathway. As shown in Figure 3A, phospho-Akt expression dramatically increased in UUO-kidneys, whilst artesunate administration significantly reduced the phosphorylation of Akt by 52%. This was further supported by densitometric analysis, which confirmed a marked decrease in the Akt phosphorylation following artesunate treatment, restoring its level close to that observed in the sham-operated control group (Figure 3B).

Akt phosphorylation is a critical step in the activation of several downstream signalling pathways including mTORC1 pathway and promotes cell proliferation, migration and survival by phosphorylating key downstream targets, including S6 and 4E-BP1 (Liu, 2011; Wang et al., 2024). Western blotting analysis showed a dramatic upregulation of phospho-S6 and phospho-4E-BP1 in UUO-kidneys, whilst artesunate treatment significantly reduced S6 and 4E-BP1 phosphorylation by 64% and 48% respectively (Figures 3A–D). Quantitative analysis of Western blot bands revealed that artesunate treatment effectively suppressed the UUO-induced activation of both S6 and 4E-BP1, indicating potent inhibition of mTORC1 downstream signalling.

Consistent with *in-vivo* findings, our *in-vitro* results showed a reduction of Akt phosphorylation in a dose-dependent manner in response to the artesunate treatment as demonstrated by Western blot. At a concentration of 60 μM for 48 h, artesunate significantly reduced phosphorylation of Akt, S6 and 4E-BP1 by 56%, 64% and 72% respectively, compared to the untreated fibroblasts, aligning with the expressions shown in the control group (Figures 3E,F). Notably, even at lower concentrations (10 and 30 μM), artesunate exerted a partial yet significant inhibitory effect on TGF-β1–induced Akt activation, further supporting its dose-dependent suppressive action on the PI3K/Akt/mTORC1signalling cascade.

Moreover, inhibition of fibroblasts proliferation was further confirmed by immunostaining of kidney sections with Ki67, a proliferation marker. Ki67-positive nuclei were scarcely detected in the sham-kidneys (Figure 3G). UUO resulted in a dramatic increase in the detection of Ki67-positive nuclei, primarily in the interstitium, whilst artesunate administration significantly reduced the number of proliferating cells by 50% (Figures 3G,H). Quantitative evaluation of Ki67-positive cells demonstrated a significant decrease in proliferating nuclei per field of view following artesunate treatment, consistent with the observed suppression of the PI3K/Akt/mTORC1 signalling pathway.

Together, these results demonstrate the ability of artesunate to downregulate the PI3K/Akt/mTORC1 signalling pathway and suppress fibroblast activation and transdifferentiation into myofibroblasts in UUO kidneys and cultured human renal fibroblasts.

### 3.4 Artesunate restored klotho expression and attenuated Wnt/β-catenin pathway in UUO-kidneys

To further investigate the inhibitory mechanisms of artesunate on kidney fibrosis in the UUO model, the Wnt/β-catenin pathway was studied. This pathway contributes to the pathogenesis of kidney fibrosis by inducing pro-fibrotic genes, EMT, ECM production and collagen deposition (Song et al., 2024; Tan et al., 2014).

**FIGURE 4:**
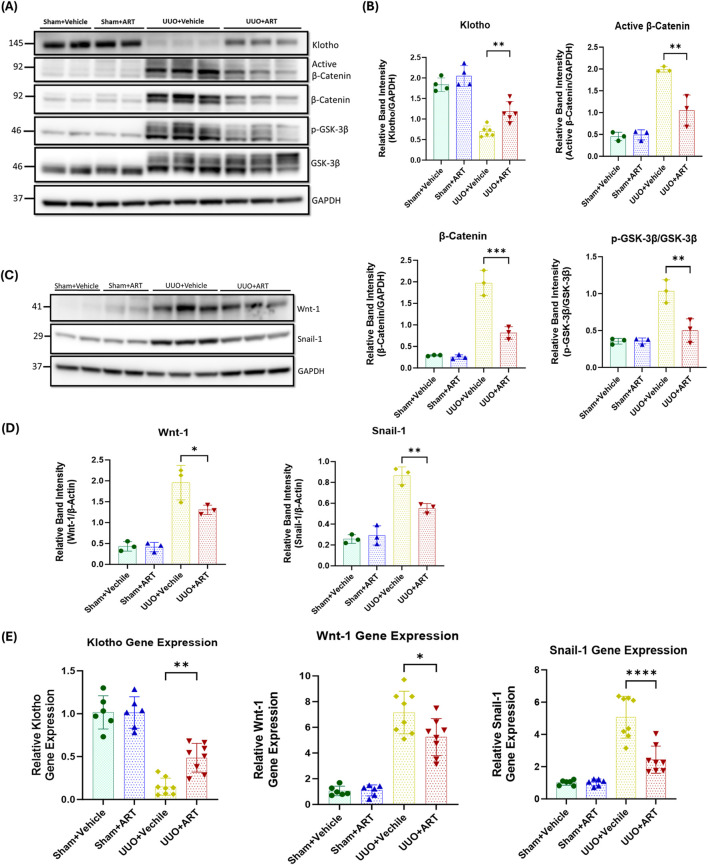
Artesunate administration restored klotho protein and attenuated Wnt/β-Catenin pathway and reduced transcription of fibrosis-related genes in UUO kidney. **(A–D)** Western blotting was performed to evaluate the expression of Klotho, Active β-Catenin, β-Catenin, p-GSK-3β, GSK-3β **(A,B)**, Wnt-1 and Snail-1 **(C,D)** proteins in UUO kidneys and control group. ImageJ software was used to quantify the blots and p-GSK-3β protein expression normalised to the total GSK-3β protein whilst the other proteins expression was normalised to GAPDH (n ≥ 3, one-way ANOVA, p ≤ 0.0339). **(E)** mRNA level of klotho, Wnt-1 and Snail-1 in UUO kidneys and sham group were quantified by using qPCR (n = 8, one-way ANOVA, p ≤ 0.022). The data are presented as the mean ± SEM from 3–8 animals per group. Statistical significance is indicated as ^ns^p ≥ 0.05, *p < 0.05, **p < 0.01, ***p < 0.001, ****p < 0.0001.

Glycogen synthase kinase-3-beta (GSK3β) is a key regulator of the Wnt/β-catenin pathway. In the presence of Wnt protein, Wnt-ligand binds to their receptor, leading to the inhibition of GSK3β activity. Inactivation of GSK3β prevents the phosphorylation and subsequent degradation of β-catenin, leading to the cytoplasmic accumulation of β-catenin which then translocation into the nucleus to induce pro-fibrotic gene expression including that for Snail (Song et al., 2024; Tan et al., 2014). Western blotting analysis showed that the artesunate administration significantly reduces Wnt-1 expression, increases GSK3β phosphorylation, reduces both β-catenin and active β-catenin and Snail-1 expression by 33%, 52%, 59%, 47%, and 36% respectively, in UUO-kidneys compared with that shown in the vehicle-treated group (Figures 4A–D).

Next, we investigated the effect of artesunate on klotho expression. Klotho acts as an antagonist of the Wnt/β-catenin pathway by directly interacting with and sequestering Wnt-ligands, thereby preventing their activation of the receptor complex (Zhou et al., 2013; Yuan et al., 2022; Zhou et al., 2022). This inhibition protects the kidney against fibrosis and helps maintain kidney function. As shown in Figures 4A,B, artesunate treatment not only reduced Wnt-1 protein expression but also partially restored klotho expression in the UUO-kidneys, whilst klotho expression was remarkably reduced in the vehicle treated UUO-kidneys.

Furthermore, gene expression assay confirmed the capability of artesunate to attenuate kidney fibrosis. Artesunate treatment significantly increased klotho mRNA and significantly reduced Wnt-1 and Snail-1 mRNA-expression in UUO-kidneys compared with that shown in vehicle-treated UUO-kidneys (Figure 4E).

Together, these results demonstrate the ability of artesunate to downregulate the Wnt/β-catenin pathway and to restore klotho expression and thereby supressing kidney fibrosis and improve kidney function in the UUO mice model.

### 3.5 Artesunate alleviated macrophages and pro-inflammatory cytokines in UUO-kidneys

**FIGURE 5:**
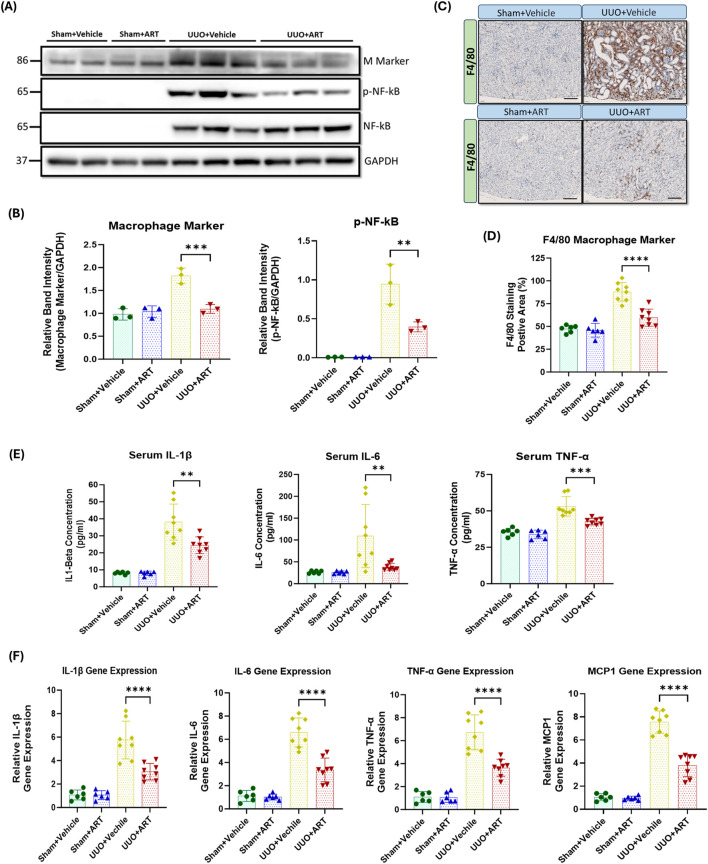
Artesunate administration suppressed macrophage marker and pro-inflammatory cytokine expression in UUO kidney. **(A,B)** the expression level of macrophage marker, p-NF-kB and total NF-Kb proteins in UUO kidneys and control group were evaluated using Western blotting technique and the blots were quantified buy using ImageJ software and normalised to the GAPDH (n = 3, one-way ANOVA, p ≤ 0.0042). **(C,D)** Immunostaining of UUO and control kidney sections for macrophage infiltration with F4/F80 at 20x magnification (scale bar 50 µm) and quantification of positively stained area as percentage of total area (n = 8, one-way ANOVA, p≤<0.0001). **(E)** The serum level of IL-1β, IL-6 and TNF-α cytokines in animals subjected to UUO surgery and sham group were measured by ELISA technique (n = 8, one-way ANOVA, p ≤ 0.0075). **(F)** mRNA level of pro-inflammatory cytokines IL-1β, IL-6, TNF-α and MCP1 in UUO kidneys and sham group were quantified by using qPCR (n = 8, one-way ANOVA, p ≤ 0.0001). The data are presented as the mean ± SEM from 3–8 animals per group. Statistical significance is indicated as ^ns^p ≥ 0.05, *p < 0.05, **p < 0.01, ***p < 0.001, ****p < 0.0001.

Inflammation is a key driver of fibrotic processes that lead to the development of CKD and ultimately, kidney fibrosis and failure (Kuang et al., 2018; Lv et al., 2018; Meng, 2019). UUO resulted in a dramatic increase in macrophage marker expression, whilst artesunate administration significantly reduced as evaluated by Western blot (Figures 5A,B). Densitometric quantification confirmed that artesunate markedly decreased macrophage marker protein expression to near basal levels, indicating effective suppression of macrophage infiltration. Immunostaining of kidney tissues with F4/80, a unique marker of murine macrophages, showed similar results. Artesunate significantly reduced F4/80 expression in UUO-kidneys compared to the UUO-kidneys from the vehicle-treated group (Figures 5C,D). Histological assessment further revealed that the number of F4/80-positive macrophages within the renal interstitium was markedly diminished following artesunate treatment, suggesting that artesunate effectively limits macrophage recruitment and accumulation in fibrotic kidneys.

Nuclear factor kappa B (NF-κB) plays a crucial role in the development of kidney fibrosis by promoting inflammation, fibroblast activation, EMT, and oxidative stress (Kuang et al., 2018; Lv et al., 2018; Meng, 2019). We therefore investigated the effect of artesunate treatment on NF-κB activation and pro-inflammatory markers expression. NF-kB was not detectable in sham-operated kidneys, whereas considerable upregulation of phospho-NF-kB was observed in UUO-kidneys. Artesunate administration significantly suppressed NF-kB phosphorylation by 58% compared to the UUO-kidneys of the vehicle-treated group (Figures 5A,B). This attenuation of NF-κB activation correlated with reduced expression of its downstream inflammatory mediators, confirming that artesunate effectively interferes with the NF-κB–dependent transcriptional response in UUO-induced injury. Similarly, artesunate administration significantly reduced serum concentrations of IL-1β, IL-6 and TNF-α by 38%, 65% and 36% respectively, in UUO mice compared to UUO vehicle-treated mice (Figure 5E). These findings were consistent with the transcriptional data and highlight artesunate’s potent anti-inflammatory effects at both the systemic and tissue levels.

Additionally, gene expression assay demonstrated the capability of artesunate to reduce inflammation in UUO-kidneys and significantly reduced IL-1β, IL-6, TNF-α and MCP1 mRNA-expression by 47%, 49%, 46% and 50% respectively, compared to the UUO-kidneys of the vehicle-treated group (Figure 5F). The concurrent reduction in cytokine protein and mRNA levels further supports that artesunate inhibits inflammatory signalling at the transcriptional level, contributing to its renoprotective and antifibrotic effects.

Overall, these results showed that the artesunate not only reduced profibrotic protein expression but was also able to downregulate pro-inflammatory cytokines and improve kidney function.

### 3.6 Artesunate increased oxidative stress and induced ferroptosis in renal fibroblasts

**FIGURE 6:**
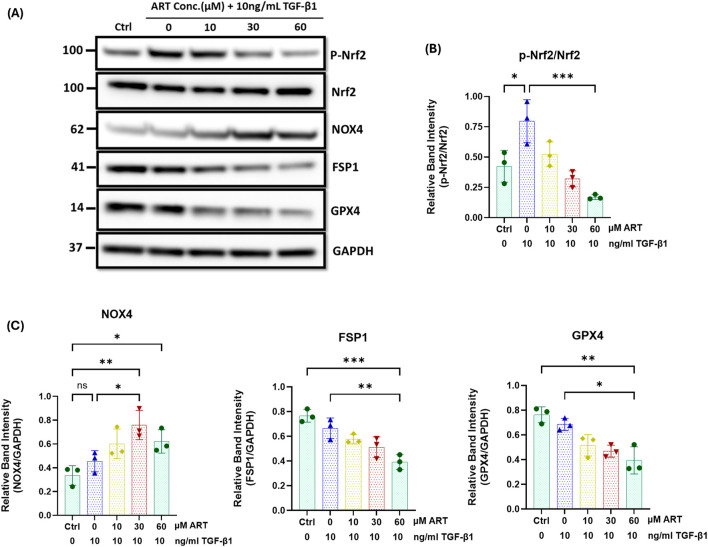
Artesunate treatment increased oxidative stress and induced ferroptosis in human primary kidney fibroblast cells. Serum starved primary human renal fibroblasts were stimulated with 10 ng/mL TGF-β1 before treatment with different concertation of artesunate ranged from 0 to 60 μM for 48 h. The cells were then lysed with RIPA buffer containing protease and phosphatase inhibitor cocktails. Protein quantity was measured using BCA method and equal protein quantity was used for Western blotting analysis. The expression level of p-Nrf2, Nrf2, NOX4, FPS1 and GPX4 **(A–C)** proteins in respond to the different concentrations of artesunate were evaluated using Western blotting technique and the blots were quantified buy using ImageJ software and p-Nrf2 normalised to the total Nrf2 protein, and all other proteins normalised to the GAPDH (n = 3, one-way ANOVA, p ≤ 0.0335). Bars represent the mean ± SEM from three independent experiments. Statistical significance is indicated as ^ns^p ≥ 0.05, *p < 0.05, **p < 0.01, ***p < 0.001, ****p < 0.0001.

Previous research shows that artesunate induces oxidative stress, elevates ROS, and triggers ferroptosis through several mechanisms (Liu et al., 2023a; Zhang et al., 2020a). Therefore, we initially investigated NADPH oxidase-4 (NOX4) expression in response to the artesunate treatment as it involves ROS production and the induction of oxidative stress. Artesunate significantly elevated NOX4 expression in renal fibroblasts, at a treatment concentration of 30 μM for 48 h, artesunate increased NOX4 expression by 40% compared to untreated fibroblasts (Figures 6A,C).

Nrf2 is another important transcription factor that plays a key role in cellular defence against oxidative stress, maintaining redox homeostasis and enhancing the activity of GPX4 and SCL7A11 to protect cells from oxidative damage (Lin et al., 2024; Tao et al., 2024; Dong et al., 2021; Ma et al., 2022). Western blotting analysis showed that the artesunate treatment gradually reduced Nrf2 phosphorylation and downregulated the expression of FSP1 and GPX4 in a dose-dependent manner in renal fibroblasts (Figures 6A–C). At a treatment concentration of 60 µM for 48 h, artesunate significantly reduced Nrf2 phosphorylation, FSP1 and GPX4 expression by 79%, 41% and 42% respectively, compared to the untreated fibroblasts.

In contrast, artesunate treatment did not significantly elevate NOX4 expression, Nrf2 phosphorylation and the expression of GPX4 and FSP1 in human renal proximal tubule cells (Supplementary Figure S3). This finding indicates that artesunate did not increase oxidative stress or induce ferroptosis in HK2 cells.

Overall, these findings suggest that artesunate selectively increases oxidative stress and induces ferroptosis in fibroblasts without affecting other kidney cells. This selective mechanism effectively inhibits fibroblasts proliferation and their transdifferentiation into myofibroblasts, a key mechanism in the pathogenesis of kidney fibrosis.

## 4 Discussion

The most important finding of this study is that artesunate treatment significantly attenuated kidney fibrosis in a UUO mouse model and induced ferroptosis in cultured human renal fibroblasts. Kidney fibrosis is characterised by excessive accumulation of ECM components and collagen within the kidney interstitium, leading to disruption of normal renal architecture, functional impairment, and ultimately progression to end stage kidney failure. The pathogenesis of renal fibrosis is very complex, involving the interplay of profibrotic growth factors, proinflammatory cytokines, oxidative stress, and dysregulated intracellular signalling pathways that drive fibroblast activation and myofibroblast differentiation (Hsieh et al., 2021; Andrikopoulos et al., 2019; Antar et al., 2023; Reiss et al., 2024).

Our study demonstrates that artesunate effectively interferes with several central fibrogenic signalling pathways, most notably the TGF-β/SMAD, PI3K/Akt/mTOR, and Wnt/β-catenin signalling cascades, thereby mitigating fibroblast activation and ECM accumulation.

TGF-β plays a pivotal role in the pathogenesis of kidney fibrosis and is consistently upregulated in CKD (Ahuja and Zaheer, 2024; Lee et al., 2024). Consequently, inhibition of the TGF-β signalling pathway remains a promising therapeutic target to mitigate the progression of CKD and delay kidney fibrosis. The therapeutic potential of artesunate and its underlying mechanisms have been investigated in this study. Our findings have demonstrated that artesunate attenuates multiple fibrogenic signalling pathways in both *in-vitro* and *in-vivo* fibrosis models.

Artesunate has been shown to mitigate TGF-β expression by modulating both canonical (SMAD-dependent) and non-canonical (SMAD-independent) signalling pathways (Liu et al., 2023a). Chen et al. reported that artesunate reduced the expression of TGF-β, SMAD3, and collagen in the type-1 diabetic rat, ultimately attenuating the TGF-β/SMAD pathway and delaying the progression of kidney fibrosis (Liang et al., 2024). Similarly, Jingyuan et al. demonstrated that artesunate inhibited fibroblast activation by reducing TGF-β expression, decreasing SMAD2/3 phosphorylation and suppressing the TGF-β/SMAD pathway in primary human ocular fibroblasts (Liu et al., 2023a). Furthermore, Jing et al. found that artesunate reduced profibrotic proteins expression including fibronectin, collagen, and α-SMA, thereby mitigating kidney fibrosis in the UUO rat model (Cao et al., 2016).

Our findings align with these observations, showing that artesunate administration in UUO mice significantly mitigated TGF-β expression and markedly attenuated the TGF-β/SMAD pathway by reducing SMAD2/3 phosphorylation. Artesunate also alleviated the expression of key fibrotic markers, including fibronectin, collagen, α-SMA and CTGF in both UUO-kidneys and renal fibroblast cell culture and effectively inhibited fibroblast proliferation and differentiation into myofibroblasts.

Additionally, SMAD3 is a key mediator of the TGF-β/SMAD pathway and plays a crucial role in promoting kidney fibrosis (Yang et al., 2021; Chen et al., 2014). As previously reported, pharmacological inhibition of SMAD3 phosphorylation in experimental kidney injury models disrupts the TGF-β/SMAD pathway and significantly reduces the fibrotic response and kidney fibrosis (Wu et al., 2015; Tang et al., 2021). Moreover, genetic ablation of SMAD3 in murine models significantly attenuates renal fibrosis (Yang et al., 2021; Zhao et al., 2016; Chen et al., 2014). Supporting these observations, our *in-vitro* study reveals SMAD3 as an essential regulator of fibroblast proliferation and myofibroblast differentiation. We observed that artesunate significantly reduced SMAD3 phosphorylation in HKF cells, disrupting the TGF-β/SMAD pathway and potentially mitigating renal fibrosis.

In addition to the canonical TGF-β/SMAD pathway, artesunate modulated several non-canonical fibrogenic signalling cascades that act synergistically to promote kidney fibrosis. The PI3K/Akt pathway is a downstream target of TGF-β signalling that significantly contributes to renal fibrosis by promoting fibroblast activation, survival and ECM synthesis. Our results demonstrated that artesunate administration ameliorated the activation of the PI3K/Akt pathway and significantly reduced Akt phosphorylation in UUO-kidneys and primary human fibroblast cultures. Activated Akt promotes renal fibrogenesis through activation of mTORC1 and suppression of GSK-3β (Wang et al., 2024; Sun et al., 2024). mTORC1 is a key downstream effector of Akt; when activated, Akt inhibits tuberous sclerosis complex 2 (TSC2), a negative regulator of mTORC1, resulting in mTORC1 phosphorylation. Consequently, active mTORC1 enhances the translation of fibrosis-related genes by phosphorylating downstream targets like S6-ribosomal protein and 4E-BP1 (Derynck et al., 2014; Di Gregorio et al., 2020; Liu, 2011; Wang et al., 2024). Our results showed that artesunate effectively alleviated PI3K/AKT/mTORC1 signalling pathway and inhibited the fibroblast proliferation and differentiation by significantly reduction of Akt, S6-ribosomal protein and 4E-BP1 phosphorylation in both UUO-kidneys and primary human renal fibroblast cell cultures. These results suggest that artesunate suppresses the PI3K/Akt/mTORC1 axis, a key profibrotic driver, thereby restoring signalling balance in the injured kidney.

Importantly, the inhibition of PI3K/Akt signalling by artesunate also exerts broader effects on other profibrotic cascades, particularly the Wnt/β-catenin pathway, through regulatory crosstalk at the GSK3β node. The Wnt/β-catenin pathway is upregulated in kidney fibrosis and exhibits crosstalk with TGF-β signalling. TGF-β overexpression enhances the expression of Wnt-ligands and β-catenin pathway components and amplifying the fibrotic response (Vallée et al., 2017; Gumede et al., 2024). We observed a significant upregulation of the Wnt/β-catenin pathway in UUO-kidneys, which was notably suppressed by artesunate administration. Our findings elucidate the mechanisms by which artesunate attenuates Wnt/β-catenin signalling in the UUO model, and we propose four potential mechanisms. Firstly, artesunate significantly reduced TGF-β expression and signalling pathway, subsequently downregulating the Wnt/β-catenin pathway. Secondly, artesunate significantly reduced Wnt expression in the UUO-kidneys, thereby attenuating the Wnt/β-catenin pathway. Thirdly, klotho acts as an antagonist of the Wnt/β-catenin pathway and is downregulated during CKD. Previous studies have reported the protective role of klotho against kidney injury and fibrosis, demonstrating that the loss of klotho exacerbates kidney injury and accelerates the progression of CKD to kidney fibrosis (Zhou et al., 2013; Yuan et al., 2022). Lili et al. reported that the loss of klotho contributes to kidney injury by derepression of the Wnt/β-catenin pathway (Zhou et al., 2013). Additionally, Qian et al. found a klotho-derived peptide (KP1) that protects the kidneys by targeting TGF-β signalling (Yuan et al., 2022). Furthermore, Wei et al. demonstrated that dihydroartemisinin, an artemisinin derivative, suppresses renal fibrosis in a mouse model by inhibiting DNA-methyltransferase activity and enhancing klotho expression (Zhou et al., 2022). We observed a significant reduction in klotho expression in UUO-kidneys. Notably, artesunate administration not only reversed this effect but also partially restored klotho in the UUO-kidneys. This restoration was associated with the inhibition of Wnt-receptor interaction, thereby attenuating the Wnt/β-catenin pathway. Finally, previous studies have demonstrated crosstalk between the PI3K/Akt and Wnt/β-catenin pathways, with GSK3β serving as a key interaction point (Fleming-de-Moraes et al., 2022). In the absence of Wnt signalling, GSK3β phosphorylates β-catenin at multiple sites, typically Ser33, Ser37, and Thr41, marking it for ubiquitination and subsequent degradation. Conversely, in the presence of activated Akt, it phosphorylates GSK3β at Ser9, thereby inhibiting its activity. Consequently, the PI3K/Akt pathway indirectly activates the Wnt/β-catenin pathway, resulting in accumulation and nuclear translocation of β-catenin, which promotes profibrotic-gene expression (Sun et al., 2024; Fleming-de-Moraes et al., 2022; Prossomariti et al., 2020; He et al., 2021; Li et al., 2021). Concordant with earlier findings, we observed a significant upregulation of p-Akt, p-GSK3β, and β-catenin in UUO-kidneys whilst artesunate administration reversed these effects.

Collectively, these results demonstrate that artesunate exerts a broad-spectrum inhibitory effect on multiple interconnected fibrogenic signalling cascades, including the TGF-β/SMAD, PI3K/Akt/mTORC1, and Wnt/β-catenin pathways, thereby limiting fibroblast activation, ECM deposition, and tissue scarring. This multi-target modulation is particularly noteworthy, as inhibition of a single pathway often fails to achieve sustained antifibrotic efficacy due to compensatory crosstalk among signalling networks.

Beyond its antifibrotic effects, artesunate also exhibited potent anti-inflammatory activity. Inflammation is a key feature of CKD and significantly contributes to disease progression, kidney damage, fibrosis, and systemic complications. Targeting persistent inflammation shows promise in improving CKD patient outcomes (Lv et al., 2018; Meng, 2019; Huang et al., 2023; Meng et al., 2014). Previous studies have reported the anti-inflammatory effects of artesunate in various models, including UUO (Kuang et al., 2018; Liang et al., 2024; Lei et al., 2021). In line with previous findings, we demonstrated that artesunate significantly reduced pro-inflammatory cytokines IL-1β, IL-6, and TNF-α concentrations, while dramatically downregulated the NF-kB signalling in UUO-kidneys. By simultaneously targeting inflammatory and fibrogenic pathways, artesunate may exert dual renoprotective actions that collectively attenuate kidney injury and fibrosis.

Finally, inducing cell death selectively in fibroblasts is a promising therapeutic strategy for treating fibrosis. Previous research demonstrated that treatment with ferroptosis inducers, such as erastin (Zhang et al., 2020a; Zhang et al., 2020b) and artemether (Wang et al., 2019) alleviates fibrosis progression in liver fibrosis mouse models by triggering ferroptosis in hepatic stellate cells. Kong et al. reported that artesunate ameliorates CCl4-induced liver fibrosis by promoting ferroptosis in activated hepatic stellate cells (Kong et al., 2019). Likewise, Jingyuan et al. found that artesunate treatment protects against ocular fibrosis by suppressing fibroblast activation and inducing mitochondria-dependent ferroptosis (Liu et al., 2023a). Consistent with previous report, our findings demonstrated that artesunate induces ferroptosis in renal fibroblasts, inhibiting both proliferation and differentiation into myofibroblasts.

The mechanism underlying the induction of ferroptosis by artesunate in fibroblasts remains unclear. However, we identified key mechanisms through which artesunate initiates and activates ferroptosis. Artesunate treatment significantly upregulated NOX4 expression, leading to an increase in oxidative stress and an elevation in ROS production. Consequently, this led to the promotion of lipid peroxidation and the depletion of key antioxidant defences, including GSH and GPX4, which normally protect cells from oxidative damage.

Moreover, artesunate disrupts another antioxidant defence system by attenuating Nrf2 signalling and reducing FSP1 expression in renal fibroblasts. These findings suggest that artesunate inhibits fibroblast proliferation through the induction of ferroptosis by impairing the cellular antioxidant defence mechanisms. In contrast, the same concentration and duration of artesunate treatment did not alter NOX4 expression, Nrf2 phosphorylation, or the expression of FSP1 and GPX4 proteins in HK2 cells. These results suggest that artesunate selectively inhibits fibroblast proliferation and induces ferroptosis specifically in renal fibroblasts, without affecting the viability or function of other kidney cells.

In conclusion, the findings of this study suggest that artesunate exhibits promising renoprotective properties that may help delay CKD progression and mitigate renal fibrosis. In a murine model of renal fibrosis, artesunate attenuated fibrotic injury by abrogating fibroblast activation, suppressing multiple profibrotic signalling pathways, and inhibiting cell proliferation. Furthermore, in cultured primary human kidney fibroblasts, artesunate induced ferroptosis and suppressed fibroblast proliferation and differentiation. Collectively, these findings indicate that artesunate may represent a potential therapeutic strategy to slow CKD progression and limit fibrotic remodelling; however, further comprehensive preclinical and clinical studies are warranted to confirm its therapeutic potential.

## Data Availability

The original contributions presented in the study are included in the article/Supplementary Material, further inquiries can be directed to the corresponding author.
